# 

**Published:** 1848-06

**Authors:** 


### CONTENTS

OF NO. VI.

### ORIGINAL COMMUNICATIONS.

### ESSAYS AND CASES.

Art. I.—On the Diseases of the United States' Army on the Rio Grande. By William G. Proctor, M.D., of Louisville, Ky. (An Inaugural Dissertation submitted to the Trustees and Medical Faculty of the University of Louisville for the Degree of Doctor of Medicine,............461

Art. II.—Removal of a large Encephaloid Tumor. By W. L. Sutton. M.D., of Georgetown, Ky. ................489

Art. III.—Case of Tubercular Phthisis, with Remarks. By John R. Buck, M.D., of Louisville, Ky................491

### REVIEWS.

Art. IV.—Principles and Practice of Surgery. By the late Geo. M'Clellan, M.D. Edited by his son John H. B. M'Clellan, M.D. Philadelphia.........................495

Art. V.—Materia Medica and Therapeutics. By Martyn Paine, M.D., Professor of the Institutes of Materia Med ica in the Uni-versity of New York....................518

Art. VI.—An Analysis of Physiology: being a condensed view of its most important Facts and Doctrines. Designed especially for the use of students. By John J. Reese, M.D., Lecturer on Materia Medica in the Medical Institute of Philadelphia; Fellow of the College of Physicians, &c. Philadelphia, J. G. Auner, 1847..........519

Art. VII.—The Young Stethescopist, or the Student's aid to Auscultation. By Henry J. Bowditch, M.D., one of the Physicians of the Massachusetts General Hospital......519

Art. VIII.—The Obstetrical Remembrancer; or Denman's Aphorisms on Natural and Difficult Parturition: the Application and Use of Instruments, &c. Augmented by Michael Ryan, M.D. First American from the ninth London edition, with additions by Thomas F. Cock, M.D., Visiting Physician to the New York Lying-in Asylum.

Memoranda on Anatomy, Surgery, and Physiology. By Mark Noble Bower, Surgeon. Corrected and enlarged by an American Physician.

Ophthalmic Memoranda respecting those diseases of the eye which are frequently met with in practice. By John Foote, Fellow of the Royal College of Surgeons.........520

Art. IX.—Lectures on the Physical Phenomena of Living Beings. By Carlo Matteuci, Professor in the University of Pisa. Translated under the superintendence of Jonathan Pereira, M.D. F.R.S., Vice President of the Royal Medical and Chirurgical Society..............521

Art. X.—On the Farcino-Glandulous Disease in Man. By Dr. A. Marchant, of Charenton, near Paris, France: Translated for this Journal, by E. Brooks, Jr., of Boston.............................................528

SELECTIONS FROM AMERICAN AND FOREIGN JOURNALS.

Naphtha in Consumpton,..........................................533

On Certain Forms of Headache,..........................................534

Recovery from a large Dose of Aconitina,..........................................538

Use of Ice in Exhausting Diseases,..........................................540

On the Food of Children,..........................................541

On the Structure and Development of the Liver,..........................................542

ORIGINAL INTELLIGENCE.

American Medical Association..........................................547

Foreign Correspondence..........................................555

Dublin Dissector—Fifth Edition..........................................555

Obituary Notices..........................................555

